# UVR8-mediated UV-B signaling and its regulation of horticultural traits: from Arabidopsis to application

**DOI:** 10.1093/hr/uhag180

**Published:** 2026-05-06

**Authors:** Huayuan Mu, Xiaoli Ma, Jigang Li, Rongcheng Lin

**Affiliations:** Biotechnology Institute, Xianghu Laboratory, Hangzhou 311231, China; State Key Laboratory of Plant Environmental Resilience, College of Biological Sciences, Frontiers Science Center for Molecular Design Breeding, China Agricultural University, Beijing 100193, China; Biotechnology Institute, Xianghu Laboratory, Hangzhou 311231, China; State Key Laboratory of Plant Environmental Resilience, College of Biological Sciences, Frontiers Science Center for Molecular Design Breeding, China Agricultural University, Beijing 100193, China; Biotechnology Institute, Xianghu Laboratory, Hangzhou 311231, China

## Abstract

Ultraviolet-B (UV-B, 280–315 nm) radiation acts as a key environmental signal that regulates plant development and acclimation responses. The UV-B photoreceptor UV RESISTANCE LOCUS 8 (UVR8) plays a central role in perceiving and transducing these signals. In the absence of UV-B, UVR8 exists predominantly as a homodimer in the cytoplasm. Upon UV-B absorption, UVR8 dissociates into active monomers that translocate to the nucleus, where they initiate downstream signaling via interactions with core regulatory components. In this review, we systematically elaborate on the UVR8-mediated UV-B signal transduction cascade and the origin and diversification of UVR8, with a focus on the regulatory effects of UV-B on the nutritional quality, stress resilience, and reproductive traits of horticultural plants. We further propose future directions for the genetic improvement of horticultural crops and the development of light-environment manipulation technologies based on the UVR8 signaling network. This review aims to provide a theoretical reference for enhancing the economic value of horticultural products and advancing green, efficient horticultural production.

## Introduction

The solar UV spectrum comprises three wavebands: UV-A (315–400 nm), UV-B (280–315 nm), and UV-C (100–280 nm). Approximately 5% of incoming UV-B radiation penetrates the atmosphere to reach the Earth’s surface [1]. High fluence of UV-B is deleterious to plant cells, causing DNA damage and reactive oxygen species (ROS) accumulation, thereby impairing cellular integrity and viability [2, 3]. In contrast, low-dose UV-B functions as a regulatory signal that orchestrates various metabolic, developmental, and defense responses, and triggers photomorphogenic programs mediated by a dedicated UV-B photoreceptor [4–7].

Horticultural plants represent economically valuable and experimentally tractable models for dissecting UV-B signaling. As an eco-friendly physical tool, UV-B shows great practical potential in both preharvest cultivation and postharvest storage, improving product quality by boosting secondary metabolite accumulation and stress tolerance [8]. Many plants, including horticultural species, sense and respond to UV-B through the conserved photoreceptor UVR8 [9, 10].

The UV-B receptor UVR8 was first identified in *Arabidopsis thaliana* [11]. In the dark or absence of UV-B, UVR8 exists mainly as a homodimer in the cytoplasm, with a minor fraction in the nucleus. Upon UV-B perception, the dimeric UVR8 dissociates into monomers and translocates into the nucleus [10, 12]. UV-B-triggered UVR8 monomerization enables its interaction with the CONSTITUTIVELY PHOTOMORPHOGENIC 1/SUPPRESSOR OF PHYA (COP1/SPA) complex, thereby regulating downstream gene expression and activating the UV-B signaling pathway. Meanwhile, REPRESSOR OF UV-B PHOTOMORPHOGENESIS (RUP) proteins interact with UVR8 to promote its re-dimerization, preventing excessive signaling activation. COP1 and RUP1/RUP2 act coordinately to maintain the dynamic homeostasis of UV-B signaling [13–16]. In the nucleus, monomeric UVR8 initiates signal transmission through direct protein–protein interactions, converting extracellular UV-B signals into intracellular physiological responses [5, 17].

This review clarifies the molecular action mechanism of AtUVR8 and its evolutionary origin. We summarize the multifaceted effects of UV-B on horticultural plants and discuss the translational potential of the Arabidopsis UVR8 interactome for improving horticultural traits. Previous UVR8 reviews have largely focused on its molecular perception and photomorphogenic regulation or summarized UVR8 signaling via protein interactions [10, 17, 18]. This review systematically integrates UV-B signaling knowledge from the model plant Arabidopsis to horticultural crops. It establishes a unified molecular framework for understanding quality formation and stress resilience in horticultural plants and lays a theoretical foundation for high-quality, efficient, and green production via precision UV-B regulation.

## Effects of UV-B on horticultural plants

Horticultural plants have high economic value, and their marketability largely depends on nutritional and visual quality, as well as stress resilience. Understanding UV-B regulation of horticultural traits is therefore practically important. Upon UV-B perception, plants activate the biosynthesis of diverse secondary metabolites, including phenolics, terpenoids, alkaloids, and glucosinolates. These compounds enhance antioxidant capacity and stress resistance of fruits, vegetables, and medicinal plants, while promoting pigmentation of fruits, leaves, and flowers, ultimately boosting commercial value [19, 20].

### UV-B enhances nutritional quality

UV-B radiation strongly induces the accumulation of phenolic and flavonoid compounds by activating the phenylpropanoid and flavonoid biosynthetic pathways, thereby elevating the nutritional quality of horticultural products. For instance, UV-B promotes the accumulation of total phenolics, flavonols, polymethoxyflavones, and stilbenes in blueberry, peach, citrus, and grape, substantially enhancing the antioxidant capacity and nutritional value of fruits [21–25]. Similarly, UV-B effectively increases the levels of anthocyanins, flavonol glycosides, apigenin, and carotenoids in vegetables, including lettuce, kale, celery, and tomato, supporting the development of functional vegetables [26–29]. UV-B enhances phenolic accumulation and antioxidant capacity in lettuce and broccoli, though often at the cost of vegetative growth and yield [30–32]. By contrast, low-dose UV-B irradiation significantly increases vitamin C, phenolics, and flavonoids in mung bean sprouts, along with enhanced antioxidant capacity, without obvious growth inhibition [33]. Precise control of UV-B dose and duration is thus critical to balancing quality improvement and yield maintenance.

At the molecular level, UV-B upregulates the expression of key enzyme genes in phenolic biosynthesis. For example, UV-B induces the expression of *Uridinediphosphate-glycosyltransferase* (*UGT*) and *O-methyltransferase* (*OMT*) genes, promoting flavonol glycoside accumulation in peach [25]. Increased polymethoxyflavones in UV-B-irradiated citrus result from an elevated expression of the methyltransferase gene *CrOMT1* [22]. *Flavone synthase I* (*FNSI*), encoding a key enzyme for apigenin biosynthesis in celery, is significantly upregulated by low-medium UV-B (0.2–0.5 W m^−2^), increasing the apigenin content, whereas high-intensity UV-B (0.8 W m^−2^) represses *AgFNSI* expression [29].

UV-B also boosts bioactive compound accumulation in ornamental and medicinal plants. In honeysuckle, chrysanthemum, and basil, UV-B markedly enhances the synthesis of caffeoylquinic acids, iridoids, chlorogenic acid, and ascorbic acid, while strengthening the antioxidant system [34–38]. Horticultural plants are important dietary sources of vitamins, and UV-B can induce vitamin biosynthesis. For example, UV-B irradiation of oyster mushrooms efficiently converts ergosterol into vitamin D₂ and D₄ without altering fatty acid profiles, highlighting promising applications [39]. In tomato fruit, UV-B regulates carotenoid metabolism through ethylene-dependent and ethylene-independent pathways, providing a theoretical basis for optimizing nutritional quality via light management [26]. Beyond secondary metabolism, UV-B also modulates photosynthesis. Lettuce shows a coordinated enhancement of photosynthetic performance and metabolic remodeling during UV-B acclimation, with increased net photosynthetic rate and flavonoid levels [40]. Moreover, low-dose, long-wavelength UV-B increases flavonoid and anthocyanin contents in red lettuce without growth suppression [41].

### UV-B improves visual quality

UV-B radiation strongly enhances pigmentation in fruits, leaves, and floral organs by specifically regulating anthocyanin biosynthesis, thereby improving the visual quality of horticultural products. In apple, peach, and blueberry, UV-B effectively induces anthocyanin synthesis, producing more intensely colored fruits [24, 25, 42]. Similarly, UV-B exposure of grape berry skin promotes the accumulation of total phenolics and flavonols and increases the proportion of highly antioxidant compounds (e.g. cyanidins, quercetin), positively affecting wine quality [21, 43]. Blue light acts synergistically with UV-B to induce anthocyanin accumulation in tomato, producing a far stronger effect than either light treatment alone [44]. Furthermore, UV-B and low temperature synergistically induce the expression of anthocyanin biosynthesis genes *chalcone synthase* (*CHS*), *anthocyanidin synthase* (*ANS*), and *flavonoid 3-O-glucosyltransferase* (*UFGT*), representing key environmental drivers of red coloration in apple peel [45]. Red light further enhances anthocyanin production when combined with UV-B [46]. Exploiting synergistic interaction between UV-B and other light wavelengths (e.g. red and blue light) thus represents a promising strategy for optimizing horticultural production.

Leafy vegetables also benefit from UV-B exposure. UV-B irradiation induces lettuce leaf reddening, mainly via an upregulated expression of core anthocyanin pathway genes *CHS*, *flavanone 3-hydroxylase* (*F3H*), and *dihydroflavonol reductase* (*DFR*), leading to the accumulation of cyanidin and trace delphinidin [28]. Elevated anthocyanin content produces a more vibrant red hue to lettuce, significantly enhancing visual quality and market competitiveness. Anthocyanin accumulation in edible tissues increases commercial value, while floral pigmentation plays a critical role in pollinator attraction. Anthocyanin synthesis in apple flowers is coordinately regulated by developmental stage and light (particularly UV-B), with transcriptional activation of *ANS* and *DFR* underlying light-induced pigmentation [47]. Likewise, UV-B is essential for anthocyanin synthesis in rose petals, and visible light applied concurrently or sequentially further enhances anthocyanin accumulation [48].

### UV-B modulates reproductive traits

UV-B radiation can improve floral traits and enhance attractiveness to pollinators. For example, UV-B exposure increases flower diameter, nectary size, and nectar volume per flower in *Malcolmia maritima*, boosting pollinator attraction and thereby improving pollination success and seed yield [49]. In *Chiloglottis orchids*, UV-B is required for the biosynthesis of the volatile pheromone chiloglottones that attract male pollinators, demonstrating a direct role of UV-B in plant–pollinator communication [50, 51]. These findings reveal a novel function of UV-B in modulating plant–pollinator interactions.

UV-B also adjusts flowering time and flower yield. UV-B significantly inhibits flowering and reduces yield in mung bean, an effect partially alleviated by triadimefon, which promotes flowering and enhances yield through multiple protective mechanisms [52]. Pollen of *Brassica rapa* is particularly sensitive to UV-B; even with increased UV-B-absorbing compounds, pollen viability declines, likely because moderate UV-B levels fail to trigger sufficient protective responses [53]. Furthermore, UV-B acts synergistically with abscisic acid (ABA) to accelerate grape berry ripening, while reducing berry size and sugar content [21]. Collectively, UV-B exerts complex, dual effects on plant reproduction: moderate UV-B can improve floral traits and pollinator attraction, whereas high-dose or prolonged exposure reduces pollen viability and causes yield loss. In practice, determining the optimal UV-B dose for specific horticultural plants is critical to achieving both quality enhancement and yield stability.

### UV-B strengthens biotic stress resistance

Powdery mildew is a major disease in greenhouse strawberry production. Studies show that UV-B radiation effectively suppresses disease incidence, with the strongest effect observed under nighttime irradiation [54]. Similarly, rose powdery mildew is effectively controlled by UV-B, which acts through a dual mechanism involving direct pathogen inhibition and induction of plant defense responses [55]. Integration of UV-B irradiation greatly reduces the frequency of fungicide applications, lowers control costs, and delays the evolution of pathogen resistance. UV-B thus represents a sustainable physical control strategy with broad potential in greenhouse production.

Alkaloids are biologically active secondary metabolites that play critical roles in plant stress resistance and defense against pests and pathogens [20, 56]. In *Catharanthus roseus*, UV-B irradiation significantly increases the content of four indole alkaloids (ajmalicine, vindoline, catharanthine, and strictosidine), accompanied by the upregulation of the key alkaloid biosynthetic gene *10-hydroxygeraniol oxidoreductase* (*10-HGO*) [57]. Similarly, UV-B rapidly induces the expression of the alkaloid biosynthetic gene *tryptophan decarboxylase* (*TDC*) and accumulation of the monoterpene indole alkaloid brachycerine in *Psychotria brachyceras* [58]. UV radiation also promotes the synthesis of dimeric alkaloids (truxillines) in *Erythroxylum novogranatense* [59].

Glucosinolates are plant defensive metabolites with toxic or repellent activity against insects, fungi, and nematodes [60]. UV-B significantly promotes glucosinolate accumulation in broccoli sprouts, especially 4-methylsulfinylbutyl glucosinolate and 4-methoxy-indol-3-ylmethyl glucosinolate. Transcriptome analysis reveals that UV-B activates defense signaling pathways associated with salicylic acid (SA) and jasmonic acid (JA) and upregulates pathogen-responsive genes [61]. UV-A-L also stimulates glucosinolate accumulation, though less effectively than UV-B, which shows superior activity in inducing both glucosinolates and polyphenolics [62].

### UV-B activates the antioxidant defense system

The balance between UV-B-induced oxidative stress and the antioxidant system is crucial for plant acclimation to UV-B stress. In recent years, the perception of UV-B has shifted from a purely stressful factor to a regulatory signal [63]. *Raphanus sativus* responds to UV-B stress by upregulating defense proteins and antioxidant enzymes, yet still shows growth inhibition and proteome disruption, indicating a complex balance between stress impacts and defensive responses [64]. Similarly, UV-B stress activates both enzymatic and nonenzymatic antioxidant systems in *Acorus calamus*; however, high doses or prolonged exposure cause oxidative damage. Low-dose UV-B-induced antioxidant responses are more favorable for plant adaptation, suggesting that *A. calamus* has considerable adaptive potential to UV-B [65].

Overall, UV-B effects are dose-dependent and species-specific. At appropriate doses, UV-B enhances plant tolerance to biotic and abiotic stresses by inducing antioxidant systems and secondary metabolite accumulation; conversely, excessive or prolonged exposure causes oxidative damage. Practical agricultural application of UV-B thus requires optimization of dose, duration, and irradiation mode to achieve disease control without compromising plant health.

UV-B radiation induces morphological adaptations and structural defenses that enhance environmental stress tolerance. UV-B exposure significantly improves low-temperature tolerance in *Rhododendron*, likely associated with UV-B-induced phenolic accumulation. These compounds act as antioxidants and membrane stabilizers, helping maintain cellular function under adverse conditions such as low temperature [66]. Cucumber also displays multiple morphological adaptations to UV-B, including altered trichome morphology and polyphenolic accumulation. Leaves become smaller and thicker, forming a compact phenotype as an adaptive adjustment to stress [67, 68].

### UV-B affects postharvest preservation

As an abiotic elicitor, UV-B effectively induces biosynthesis of various secondary metabolites in fruits and vegetables, making it a promising physical postharvest treatment widely used for produce preservation and quality maintenance. UV-B treatment significantly increases ascorbic acid, lycopene, and β-carotene contents in tomato peel and flesh, while moderately improving flesh color [69]. Similarly, it markedly elevates anthocyanin content and enhances key enzyme activities of the phenylpropanoid pathway in chrysanthemum [70]. Studies on peach and nectarine further show that UV-B can increase flavonol and anthocyanin contents in the peel of certain cultivars, accompanied by the upregulation of related biosynthetic genes, including *phenylalanine ammonialyase* (*PAL*), *4-coumarate: coenzyme A ligase* (*4CL*), and *CHS* [71]. However, UV-B-induced phenolic accumulation shows a strong cultivar and dose dependency. For instance, phenolic content in peach ‘Babygold 7’ decreases after UV-B treatment, with the most biosynthetic genes downregulated [71]. An appropriate UV-B dose (3.6 kJ m^−2^) effectively delays grape berry softening and promotes the accumulation of flavanols, anthocyanins, and resveratrol, whereas a high dose (7.2 kJ m^−2^) causes photodamage, accelerates chlorophyll degradation, and activates defense enzyme systems [72]. These findings highlight the need to optimize UV-B treatment conditions for different species and cultivars in practical use.

**Figure 1:**
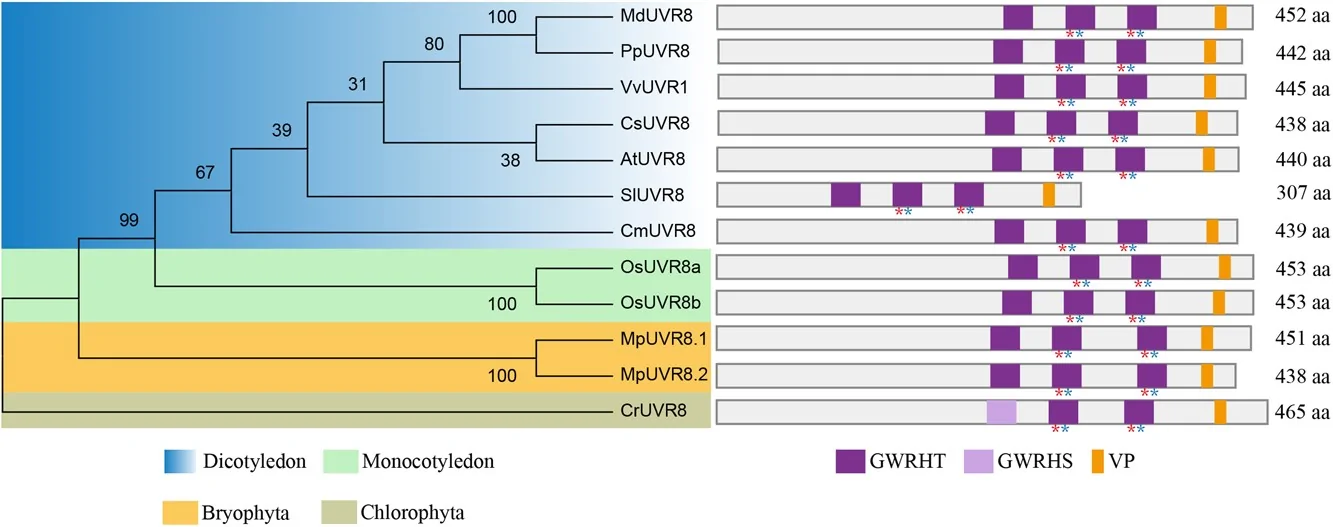
The phylogenetic tree (left panel) and corresponding conserved motifs (right panel) of UVR8.

In addition to promoting phenolic synthesis, UV-B benefits postharvest storage by enhancing disease resistance, chilling tolerance, and delaying senescence. Ochratoxin A (OTA), a highly toxic secondary metabolite produced by *Aspergillus* and *Penicillium* fungi, contaminates many agricultural products and threatens human and animal health. UV-B exposure strongly inhibits OTA production by downregulating key OTA biosynthetic genes, especially *otaA* and *otaR1*, and can directly degrade preformed OTA, supporting its postharvest preservation potential [73]. Preharvest UV-B pretreatment of tomato and mango alleviates chilling injury during low-temperature storage, delays ethylene release and fruit softening, and preserves better nutritional quality [74, 75]. Furthermore, blueberry fruits treated with a combination of UV-B and UV-C show reduced decay and weight loss, along with increased anthocyanin and total phenolic content and enhanced antioxidant capacity [24].

As an eco-friendly physical treatment, UV-B effectively induces phenolic biosynthesis in horticultural plants and plays multifunctional roles in postharvest preservation, including disease resistance, chilling tolerance, and quality retention. UV-B acts as a signal molecule that positively regulates photomorphogenesis and phenolic accumulation, a process mainly mediated by the specific UV-B photoreceptor UVR8. How UVR8 perceives UV-B signal and initiates transduction to ultimately regulate diverse biological processes in horticultural plants requires further investigation. Future research should also clarify the network mechanisms underlying UV-B signaling and metabolic regulation across species and establish cultivar- and dose-specific optimization systems to promote the practical application of UV-B in green postharvest management.

## Origin and evolution of UVR8

Since UVR8 was first discovered and systematically characterized in Arabidopsis, its homologs have been identified in an expanding range of plant species, including green algae, bryophytes, lycophytes, and angiosperms. Phylogenetic analyses indicate that UVR8 originated in the green algal clade of Viridiplantae, representing the earliest emergence of UVR8 in the plant kingdom [76, 77]. UVR8 proteins are relatively conserved across species, with a clear divergence among green algae, bryophytes, dicots, and monocots. Higher sequence conservation is observed in the tryptophan triad (GWRHT motifs) and VP motif within the C27 domain, while variations exist in *Chlamydomonas reinhardtii* and *Solanum lycopersicum* (Fig. 1). UVR8 is present as a single-copy gene in Arabidopsis, green algae, and some early-diverging plant lineages; however, certain species such as moss (*Physcomitrella patens*), rice (*Oryza sativa*), and bean carry two copies [76, 78, 79]. Whether multiple UVR8 copies in some species reflect functional redundancy or divergence during environmental adaptation requires further in-depth study. Seagrass (*Zostera marina*), one of the few angiosperms that have re-colonized marine habitats, has completely lost the UVR8 gene, likely associated with attenuated UV-B radiation in submerged environments, revealing genomic evolution linked to environmental adaptation [80].

A phylogenetic tree was reconstructed with the neighbor-joining method in MEGA12 software. Bootstrap analysis was conducted with 1000 replicates. Evolutionary distances were computed using the Poisson correction method and are expressed as the number of amino acid substitutions per site. The critical tryptophan residues for UV-B absorption (red asterisks), arginine residues critical for the stable salt-bridge network (blue asterisks), conserved GWRHT, and VP motifs were indicated: At, *A. thaliana*; Cm, *C. morifolium*; Cr, *C. reinhardtii*; Cs, *Cucumis sativus*; Md, *Malus domestica*; Mp, *Marchantia polymorpha*; Os, *O. sativa*; PpUVR8, *Prunus persica*; Sl, *S. lycopersicum*; Vv, *Vitis vinifera*.

## UVR8-mediated UV-B signaling

UVR8 contains core functional regions: key tryptophan residues for UV-B absorption, a salt-bridge network that stabilizes the dimer state, a β-propeller domain, and the C27 domain responsible for interactions with regulatory partners ([7, 81–86]; Fig. 1). UV-B absorption by UVR8 tryptophans disrupts the salt-bridge network, triggering rapid dissociation of dimers into active monomers in a COP1-independent manner [7, 81]. Once in the nucleus, monomeric UVR8 interacts with COP1 in a UV-B-dependent manner [4, 7, 86]. This interaction promotes UV-B-triggered photomorphogenesis by modulating transcription factors such as ELONGATED HYPOCOTYL 5 (HY5) and its regulatory network [10, 86]. Signaling attenuation is mediated by RUP1 and RUP2, which bind UVR8 independently of UV-B and promote monomer re-dimerization [15, 86]. The opposing activities of COP1 and RUP1/2 establish a dynamic equilibrium that fine-tunes the duration and amplitude of UV-B signaling [14, 86, 87].

**Figure 2:**
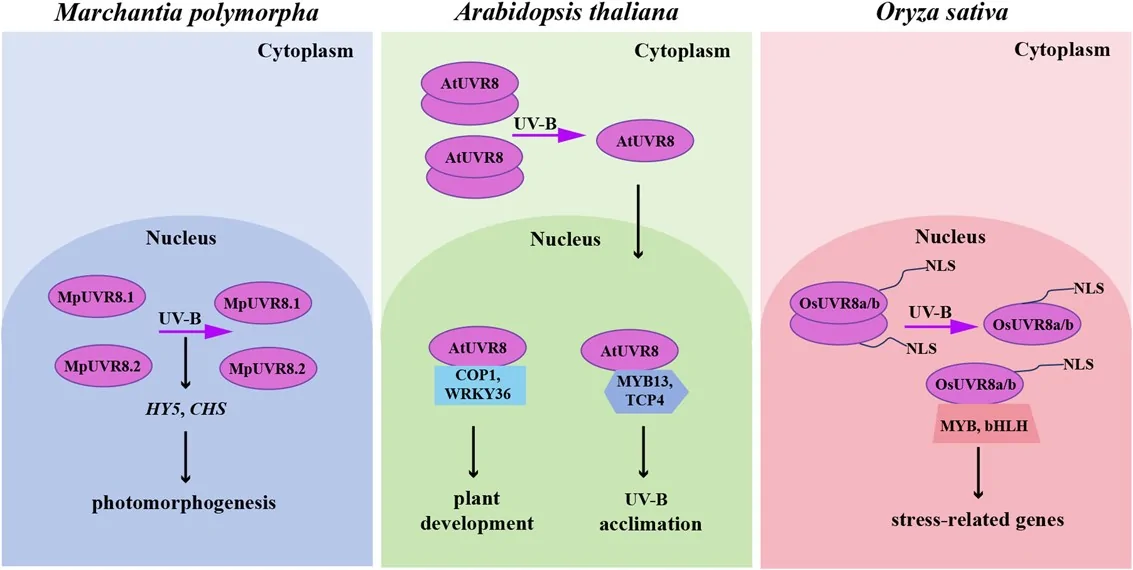
Response of UVR8 proteins from different species to UV-B.

UVR8 protein from Euphrates poplar, grape, chrysanthemum, and apple functions similarly to AtUVR8, confirming evolutionary conservation of the UVR8-COP1-HY5 module in dicots [88–91]. Nevertheless, bryophytes (e.g. liverwort *M. polymorpha*), dicots (e.g. Arabidopsis), and monocots (e.g. rice) display distinct UV-B responses. Due to weaker dimer stability, MpUVR8.1 and MpUVR8.2 exist mainly as monomers and show constitutive nuclear localization regardless of UV-B. In contrast, AtUVR8 and OsUVR8a/b are dimeric in the absence of UV-B and monomerize upon UV-B perception. A key distinction is subcellular localization: AtUVR8 is distributed throughout the cell and translocated to the nucleus specifically in response to UV-B, whereas OsUVR8s show constitutive nuclear localization mediated by a C-terminal nuclear localization signal (NLS), independent of UV-B or OsCOP1. Functionally, UV-B perception in liverwort and Arabidopsis upregulates *HY5* and *CHS*, promoting photomorphogenesis, whereas UV-B-induced monomerization of OsUVR8s facilitates interactions with MYB/bHLH transcription factors and activates stress-related gene expression ([10, 78, 79]; Fig. 2). Overall, UVR8 displays both functional conservation and species-specific characteristics, reflecting plant adaptation to diverse ecological niches and environmental pressures.

*A. thaliana* and *M. polymorpha* each contain a single UVR8 gene, whereas *O. sativa* harbors two UVR8 genes (OsUVR8a and OsUVR8b). However, MpUVR8 produces two transcript variants (MpUVR8.1 and MpUVR8.2) via alternative splicing. Due to their weaker stability, MpUVR8.1 and MpUVR8.2 exist predominantly as monomers even in the absence of UV-B and exhibit constitutive nuclear localization. In contrast, AtUVR8 and OsUVR8a/b exist as dimers without UV-B and monomerize upon UV-B exposure. AtUVR8 is distributed in both the cytoplasm and nucleus, and UV-B induces its nuclear translocation. Unlike AtUVR8, OsUVR8s display constitutive nuclear localization dependent on their C-terminal NLS, independent of UV-B or OsCOP1. In *M. polymorpha* and *A. thaliana*, UVR8 perceives UV-B signaling, leading to the increased expression of *HY5* and *CHS* and promoting photomorphogenesis. In rice, UV-B-induced monomerization of OsUVR8s facilitates their interaction with MYB and basic helix–loop–helix (bHLH) transcription factors, subsequently upregulating stress-related gene expression.

## Applications of the Arabidopsis UVR8 interactome in horticultural trait research

**Figure 3:**
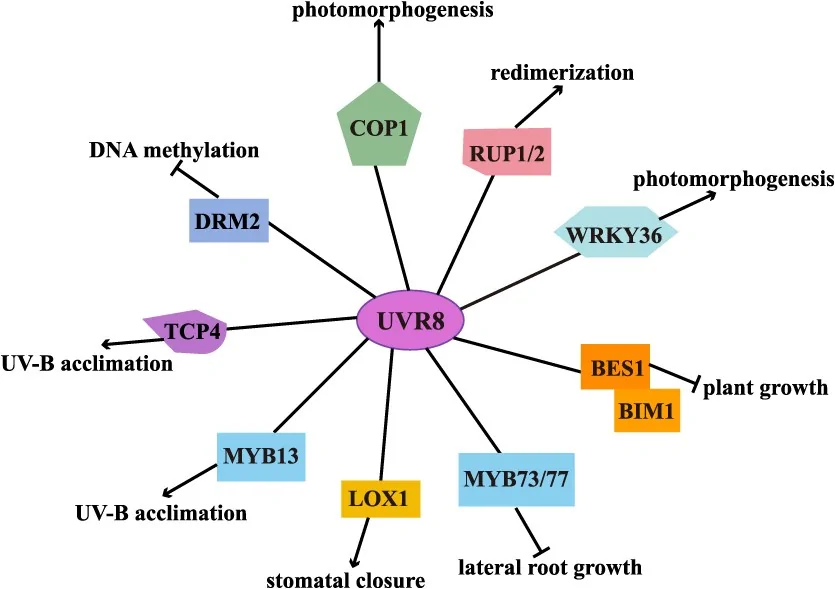
UV-B signal transduction processes mediated by UVR8 in Arabidopsis. UVR8 interacts with various proteins (e.g. COP1, RUP1/2, WRKY36, BES1, MYB73/77, LOX1, MYB13, TCP4, DRM2) to participate in distinct biological processes.

Studies of plant UV-B signal transduction began in Arabidopsis, establishing a core signaling framework centered on the UV-B photoreceptor. The UVR8-COP1-HY5 module constitutes the central hub of UV-B signaling. In darkness or the absence of UV-B, COP1 acts as an E3 ubiquitin ligase that mediates HY5 degradation. Upon UV-B activation, UVR8 interacts with COP1, reducing COP1-mediated HY5 degradation, thereby stabilizing HY5 and promoting its accumulation [5, 92, 93]. HY5 directly activates the expression of anthocyanin biosynthetic genes and MYB transcription factors, regulating downstream signaling cascades [94]. This module is highly conserved in horticultural plants. For example, in apple, MdHY5 directly binds to the G-box element in the *MdMYB10* promoter to activate its expression [95]. In tomato, SlUVR8 enhances plant adaptation to UV-B by regulating *SlHY5* and *SlCHS* expression [96]. CsbZIP1 (an HY5 homolog) in tea plants not only directly activates *CsMYB75* but also represses the negative regulator *CsMYBL2* by inducing *CsmiR858a* expression [97], ultimately promoting anthocyanin accumulation. These findings confirm the conservation of the UVR8-mediated UV-B signaling pathway in horticultural plants. Recent advances in the Arabidopsis UVR8 interactome (Fig. 3, Table 1) provide new insights and research paradigms for horticultural plant studies. Translating UVR8-interacting proteins and their regulatory mechanisms identified in Arabidopsis to the research on key horticultural traits will be a key strategy to uncover molecular mechanisms underlying pigmentation, nutritional quality, and stress tolerance.

UV-B induces secondary metabolite biosynthesis in horticultural plants, with MYB and WRKY transcription factors playing critical regulatory roles in the phenylpropanoid pathway [107, 108]. Whether UVR8 in horticultural plants, like AtUVR8, interacts with MYB and WRKY transcription factors to relay UV-B signal into secondary metabolic pathways requires further investigation [100, 102, 103].

Studies in Arabidopsis have revealed the integration of UVR8 with multiple hormone signaling pathways. For example, UVR8 interacts with BRI1-EMS-SUPPRESSOR 1 (BES1)/BES1-INTERACTING MYC-LIKE 1 (BIM1) to suppress brassinosteroid signaling, coordinating growth and defense [101]. It also interacts with MYB73/MYB77 to inhibit auxin signaling and regulate lateral root development [102] and with MYB13 to rebalance auxin signaling and flavonoid metabolism [103]. These integration nodes provide entry points for dissecting multi-trait coordination in horticultural plants. For instance, the trade-off between fruit color and size in apple, metabolic partitioning between catechins and anthocyanins in tea, and coordination between phenolic accumulation and ripening in grape may involve the crosstalk between UVR8 and hormone signaling.

Furthermore, UVR8 interaction with TEOSINTE BRANCHED1, Cycloidea, and PCF 4 (TCP4) activates JA signaling [105], while interaction with LIPOXYGENASE 1 (LOX1) activates SA signaling [106]. These mechanisms offer new perspectives for studying stress tolerance in horticultural plants. For instance, the UV-B-mediated suppression of powdery mildew in strawberry and rose [54, 55] may involve the UVR8-TCP4-JA or UVR8-LOX1-SA pathways.

The interaction between UVR8 and DOMAINS REARRANGED METHYLTRANSFERASE 2 (DRM2) in Arabidopsis reveals a direct link between UV-B signaling and DNA methylation [104]. This interaction inhibits DRM2 chromatin binding, altering the methylation status at specific genomic loci. This finding suggests that UV-B may shape long-term adaptability in horticultural plants through epigenetic regulation. In horticultural production, phenomena such as rootstock–scion crosstalk, graft-induced trait changes, and seasonal responses in perennial fruit trees may involve epigenetic mechanisms. As a manageable physical treatment, UV-B irradiation could induce stable trait modifications via epigenetic modules such as the UVR8-DRM2. Research in this area remains largely unexplored in horticultural plants and represents a key future direction.

## Conclusions and prospects

Over the past two decades, major progress has been made in understanding Arabidopsis UVR8. The current model positions UVR8 as a dynamically light-regulated nucleocytoplasmic shuttle protein. Its nuclear activity is governed by a coordinated interplay of UV-B-induced monomerization, passive nuclear diffusion, COP1-mediated nuclear retention, and RUP-driven re-dimerization. Spatially distinct interactions between UVR8 and COP1 orchestrate a precisely timed signaling cascade that converts a transient UV-B stimulus into a sustained photomorphogenic response, exemplifying the sophisticated regulatory strategies plants use to adapt to their light environment.

Research on the UVR8 mechanism and function in non-Arabidopsis species remains relatively limited. Studies on UVR8-mediated flowering have focused mainly on Arabidopsis [109, 110], with direct evidence in horticultural plants still scarce, and its role in fruit ripening remains largely unexplored. Recent transcriptomic data showing the activation of the UVR8 pathway in UV-B-treated peach fruit [111] suggest broader functional relevance. Investigating UVR8 function in flowering time and postharvest ripening in horticultural species thus represents a promising, understudied direction, with potential to both expand the understanding of UVR8 signaling and guide light-based strategies for improving crop yield and quality. Future studies can leverage approaches and concepts established in Arabidopsis to explore molecular functions of UVR8 in a wider range of plant species, especially horticultural crops.

**Table 1:** UVR8 interacting proteins in *A. thaliana*.

**Protein**	**Binding domain of UVR8**	**Binding domain of interacting protein**	**Interacting condition**	**Function**	**Reference**
**COP1**	C27 domain, core domain	WD40 domain	UV-B dependent	UVR8-COP1 interaction reduces the ubiquitination and degradation of HY5 by COP1-SPA complex. COP1 promotes the nuclear accumulation of UVR8	Favory *et al.* [4] Huang *et al.* [98] Yin *et al.* [16]
**RUP1/2**	C27 domain, core domain	WD40 domain	UV-B independent	UVR8-RUP1/2 interactions induce re-dimerization of UVR8 and suppress the nuclear accumulation of UVR8	Gruber *et al.* [99] Qian *et al.* [14]
**WRKY36**	C terminus	C-terminal DNA-binding domain	UV-B independent	UVR8-WRKY36 interaction relieves the transcriptional repression of HY5 by WRKY36, leading to increased expression of HY5 and photomorphogenesis	Yang *et al.* [100]
**BES1/BIM1**	C terminus	BIN domain of BES1, C terminus of BIM1	UV-B independent	UVR8-BES1/BIM1 interactions inhibit the expression of BES1-targeted growth-related genes, ultimately suppressing plant growth	Liang *et al.* [101]
**MYB73/77**	N and C termini	N terminus	UV-B dependent	UVR8 strongly inhibits the binding activity of MYB73/MYB77 to auxin-responsive genes and inhibits lateral root growth	Yang *et al.* [102]
**MYB13**	/	C-terminal SG domain	UV-B dependent	UVR8 inhibits MYB13 binding to SAUR promoters while promoting its binding to flavonoid biosynthesis gene promoters, leading to downregulation of auxin-related genes and upregulation of flavonoid biosynthesis genes	Qian *et al.* [103]
**DRM2**	C terminus, core domain	UBA domain	UV-B independent	UVR8-DRM2 interaction prevents the binding of DRM2 on chromatin and inhibits the DNA methylation mediated by DRM2	Jiang *et al.* [104]
**TCP4**			UV-B dependent	UVR8-TCP4 interaction enhances TCP4’s binding to the LOX2 promoter and its expression and thus activates the JA and anthocyanin biosynthesis pathways and plant tolerance to UV-B	Li *et al.* [105]
**LOX1**	C60 domain	C1 domain	UV-B independent	UVR8-LOX1 interaction enhances the activity of LOX1, which induces oxylipin production, and then, oxylipins activate the SA signaling pathway, leading to stomatal closure	Liu *et al.* [106]

In Arabidopsis, UVR8 interacts with multiple proteins to regulate UV-B-triggered biological processes, including photomorphogenesis, leaf and root development, stomatal closure, and UV-B acclimation. However, studies of UVR8 biological functions in other species remain scarce, largely limited to complementation assays in the Arabidopsis UVR8 mutant. A substantial knowledge gap thus exists regarding both the interacting partners and biological functions of UVR8 in other non-model plants. Elucidation of the Arabidopsis UVR8 interactome has provided a clear framework and abundant candidate gene resources for studying UV-B signal transduction in horticultural plants. Through a research pipeline of homologous gene cloning, interaction validation, functional characterization, and trait association, fundamental discoveries from Arabidopsis can be efficiently translated into molecular mechanisms governing trait regulation in horticultural crops. Future research should further focus on species-specific signaling pathways, epigenetic regulatory mechanisms in perennial horticultural plants, and integrated regulatory networks mediating crosstalk between UV-B signaling and multiple endogenous/exogenous factors. These investigations will lay a solid theoretical foundation for applying precision light regulation technologies in horticultural production.
